# Enhancement of the Norfloxacin Antibiotic Activity by Gaseous Contact with the Essential Oil of *Croton zehntneri*

**DOI:** 10.4103/0975-1483.71625

**Published:** 2010

**Authors:** HDM Coutinho, EFF Matias, KKA Santos, SR Tintino, CES Souza, GMM Guedes, FAD Santos, JGM Costa, VS Falcão-Silva, JP Siqueira-Júnior

**Affiliations:** *Laboratory of Microbiology and Molecular Biology and Center of Exacts and Natural Sciences, Federal University of Paraba, Joo Pessoa (PB)*; 1 *Laboratory of Research in Natural Products, Center of Biological Sciences and Health, University of the Region of Cariri, Crato (CE), Brazil*; 2 *Laboratory of Genetics of Microrganisms, Department of Molecular Biology, Center of Exacts and Natural Sciences, Federal University of Paraba, João Pessoa (PB)*

**Keywords:** *Croton zehntneri*, gaseous contact, multidrug resistant pathogens, minimal inhibitory dose, *Staphylococcus aureus*

## Abstract

This is the first on the modulation of norfloxacin antibiotic activity by the volatile compounds of an essential oil. We report the chemical composition and antibiotic modifying activity of the essential oil extracted from the leaves of *Croton zehntneri* Pax et Hoffm (variety estragole), using the minimal inhibitory dose method and gaseous contact. The leaves of *Croton zehntneri* Pax et Hoffm (Euphorbiaceae) were subjected to hydrodistillation, and the essential oil extracted was examined with respect to the chemical composition, by gas chromatography-mass spectrometry (GC/MS), and to inhibitory activity of efflux pump by gaseous contact. The main component of the essential oil of *C. zehntneri* was estragole (76,8%). The gaseous components of the oil enhanced the inhibition zone of norfloxacin in 39,5%. This result shows that this oil influences the antibiotic activity of norfloxacin, possibly affecting the bacterial NorA efflux system, and may be used as an adjuvant in the antibiotic therapy of multidrug resistant pathogens.

## INTRODUCTION

Modification of the antibiotic activity agents is done by compounds that enhance the activity of antibiotics, and plants are a rich source of these compounds.[1] Essential oils are natural, complex, multi-component systems composed mainly of terpenes, in addition to some other non-terpene components. [2] Strong *in vitro* evidence indicates that essential oils can act as antibacterial agents against a wide spectrum of pathogenic bacterial strains including *Listeria monocytogenes, L. innocua, Salmonella typhimurium, Escherichia coli* O157:H7, *Shigella dysenteria, Bacillus cereus, *Staphylococcus aureus**, and *Salmonella typhimurium.*[3] *Croton zehntneri* Pax et Hoffm is an aromatic plant, native to Northeastern Brazil, where it is popularly called ’Canela de Cunhã,’ and is principally used in folk medicine as a sedative, as an appetite-stimulating antianorexigen, and for the relief of gastrointestinal disturbances.[4,5] Despite this, little pharmacological investigation has been carried out on the effects of *C. zehntneri*. Published research on plant extracts has focused primarily on the central nervous system and on muscles.[6–9] The chemical composition is essentially characterized by a large percentage of oxygenated sesquiterpenes.[10] However, four varieties of *C. zehntneri* can be distinguished, based on their main component: anetol, eugenol, methil-eugenol, or estragole.[11]

In this article, we report the chemical composition and antibiotic modifying activity of the essential oil extracted from the leaves of *Croton zehntneri* Pax et Hoffm (variety estragol), using the minimal inhibitory dose method and gaseous contact.

## MATERIALS AND METHODS

### Plant material and essential oil extraction

Leaves of *Croton zehntneri* Pax et Hoffm (Euphorbiaceae) were collected in Crato county, Ceará State, Brazil. The plant material was identified and a voucher specimen was deposited under the number 1619 at the Herbarium ’Dárdano de Andrade Lima’ of Universidade Regional do Cariri - URCA. Fresh leaves (1.750 g) of *Croton zehntneri* were collected from the Medicinal and Aromatic Plant Garden at the Pimenta Campus of the Regional University of Cariri (URCA), Crato, CE, Brazil, in March 2008. The leaves were triturated and extracted by hydrodistillation for two hours using a Clevenger-type apparatus. The oil was collected, dried using anhydrous sodium sulfate, and subsequently stored under low light conditions at < 10°C, until analysis.

### Gas liquid chromatography coupled mass spectometry (GC/MS) analysis

The analysis of volatile constituents was carried out in a Hewlett-Packard GC/MS, model 5971, using the non-polar fused silica column DB-1 (25 cm × 0.20 mm i.d., 0.25 μm film), eluted with helium gas at 8 mL/minute, with a split mode. Injector and detector temperatures were set to 250°C and 200°C, respectively. The column temperature was programmed from 50°C to 180°C at 4°C/minute, and then from 180°C to 280°C at 20°C/minute. Mass spectra were recorded from 30 to 450 m/z, with an electron beam energy of 70 eV. The individual components were identified by computer MS library searches, using retention indices as a pre-selection routine, and visual inspection of the mass spectra from the literature, for confirmation, as well as by visually comparing standard fragmentation with that reported in the literature.

### Strain

The strain used was *Staphylococcus aureus* SA 1199B, which overexpressed the *norA* gene encoding the NorA efflux protein, which extruded fluoroquinolones as norfloxacin. The strain, kindly provided by Professor Simon Gibbons (University of London), was maintained in Blood Agar Base (Difco Laboratories Ltda., Brazil) slants, and prior to use, the cells were grown overnight at 37°C in a brain heart infusion broth (BHI- Difco Laboratories Ltda., Brazil).

Antibiotic modifying activity

The antibiotic modifying activity of the volatile components of the essential oil from *Croton zehntneri* was assayed using gaseous contact. An amount of 50 μg of oil was dissolved in 50 μl of DMSO (1:1). A twofold dilution series of this essential oil solution was prepared: 50, 25, 12.5, 6.25, 3.125, and 1.562 μg of oil. Petri dishes with nutrient agar (Difco) were inoculated with 10^5^ CFU/ml, using the spread plate method. A volume of 100 μl of the dilution with 12.5 μg (MID = 0,25 mg/L air) of the oil was placed inside the lid of a Petri dish. The plates were incubated at 37°C for 24 hours, with the lid down, to avoid any contact between the solution and the bacterium. In these plates, antibiotics disks with norfloxacin were used to determine the changes in the inhibition zone diameter of *S. aureus* 1199B. Plates without the essential oil and with DMSO alone were used as control.[12]

## RESULTS AND DISCUSSION

In Table 1, the chemical characterization of the essential oil of *C. zehntneri* (EOCZ) shows a high content of mono- and sesquiterpenes, mainly estragol, representing 76,8% of the content.

**Table 1:** Chemical composition of the essential oil of *C. zehntneri*

Compounds	Retention time (Min.)	Oil (%)
Estragole	28,8	76,8
1,8 Cineole	21,8	7,0
Eugenol	35,0	5,3
Myrcene	19,6	4,4
Biciclogermacrene	37,7	1,7
Beta-ocimene	22,2	1,6
Sabinene	19,2	0,61

In Table 2, the antibiotic activity of norfloxacin was enhanced in 39,5%, in the presence of the volatile compounds from the essential oil. Synergy research aims to find the scientific reasons to the superiority of many herbal drug extracts as compared to the single constituents thereof.[12]

The mechanisms by which essential oils can inhibit microorganisms involve different modes of action, and in part may be due to their hydrophobicity. As a result, they get partitioned into the lipid bilayer of the cell membrane, affecting the respiratory chain and the energy production,[13] rendering it more permeable for the uptake of antibiotics and leading to leakage of vital cell contents. Several components of essential oils, such as, thymol and carvacrol may act as membrane permeabilizers, enhancing the intake of antibiotics.[14] Bacterial efflux pumps are energy-dependent (ATP) pumps that can be affected by these permeabilizers; besides bacterium species that constitutively express such transporters are intrinsically least affected by various antibiotics.[15]

**Table 2:** Enhancement of the antibiotic activity by the volatile compounds of *Croton zehntneri*, by inhibition of efflux pump

Treatments	*S. aureus* 1199B (mm ± SD %)	
	Norfloxacin	Enhancement (%)
No treatment	8,6 ± 1,6	-
DMSO	8 ± 0,6	-
EOCZ (MID 0,25 mg/L air)	12 ± 1	39,5

EOCZ: Essential oil of *Croton zehntneri*,SD: Standard deviation

This strategy is called ‘herbal shotgun’ or ‘the synergistic multi-target effect’ and refers to the use of herbals and drugs in a multitargeted approach, due the fact that mono- or multi-extract combinations not only affect a single target, but several ones, cooperating in an agonistic-synergistic way. This approach is not exclusive to extract combinations, and combinations between single natural products or extracts with chemosynthetics or antibiotics is also possible.[12,16–19]

Individual compounds of essential oils from *Melaleuca leucodendron and Ocimum gratissimum* presented synergism with several antibiotics by direct contact,[20] but only by using the direct contact oil-bacteria. Using gaseous contact, the modifying antibiotic activity of *C. zehntneri, Zanthoxylum articulatum* and *Vanillosmopsis arborea* was demonstrated against gentamicin.[12] Due the results indicated in this study, we can show the possibility of using vapor from the essential oil of *C. zehntneri* and variety estragole, to enhance the antibiotic activity of commonly used antibiotics, such as, norfloxacin.

## CONCLUSION

Several reports indicate different antibiotic combinations assayed *in vitro* and applied in the clinics, but combinations of natural products and synthetic drugs have not been reported. The results obtained in this investigation suggest that the volatile compounds of the essential oil of *C. zehntneri* may modulate the antibiotic activity of norfloxacin and may be a source of metabolites with an antibacterial modifying activity, to be used as an adjuvant to antibiotic therapy, against MDR pathogens. In the future, *in vivo* assays will be selected and constructed to verify if this result can be reproduced in the organism, using the inhalation form, and if positive, the next step will be clinical tests and trials.
